# N-butyl-β-carboline-3-carboxylate (β-CCB) systemic administration promotes remyelination in the cuprizone demyelinating model in mice

**DOI:** 10.1038/s41598-024-64501-x

**Published:** 2024-06-18

**Authors:** Fidel Vélez-Uriza, Rainald Pablo Ordaz, Edith Garay, Abraham J. Cisneros-Mejorado, Rogelio O. Arellano

**Affiliations:** Instituto de Neurobiología, Laboratorio de Neurofisiología Celular, Universidad Nacional Autónoma de México, Boulevard Juriquilla 3001, Juriquilla Querétaro, C.P. 76230 México

**Keywords:** Cuprizone, GABA, β-Carboline, Oligodendrocyte, Remyelination, MRI, Cell biology, Neuroscience

## Abstract

Demyelination is generated in several nervous system illnesses. Developing strategies for effective clinical treatments requires the discovery of promyelinating drugs. Increased GABAergic signaling through γ-aminobutyric acid type A receptor (GABA_A_R) activation in oligodendrocytes has been proposed as a promyelinating condition. GABA_A_R expressed in oligodendroglia is strongly potentiated by n-butyl-β-carboline-3-carboxylate (β-CCB) compared to that in neurons. Here, mice were subjected to 0.3% cuprizone (CPZ) added in the food to induce central nervous system demyelination, a well-known model for multiple sclerosis. Then β-CCB (1 mg/Kg) was systemically administered to analyze the remyelination status in white and gray matter areas. Myelin content was evaluated using Black-Gold II (BGII) staining, immunofluorescence (IF), and magnetic resonance imaging (MRI). Evidence indicates that β-CCB treatment of CPZ-demyelinated animals promoted remyelination in several white matter structures, such as the *fimbria*, *corpus callosum*, *internal capsule*, and *cerebellar peduncles*. Moreover, using IF, it was observed that CPZ intake induced an increase in NG2^+^ and a decrease in CC1^+^ cell populations, alterations that were importantly retrieved by β-CCB treatment. Thus, the promyelinating character of β-CCB was confirmed in a generalized demyelination model, strengthening the idea that it has clinical potential as a therapeutic drug.

## Introduction

Demyelinating diseases represent an important percentage of disabling disorders in the nervous system (NS). Finding potent and specific promyelinating drugs is necessary to develop strategies that could allow for effective clinical treatments. Recent research has proposed that γ-aminobutyric acid (GABA) signaling is involved in myelination control^1–4^ and that GABARs, both GABAR type A (receptor-channel, GABA_A_R) and GABAR type B (coupled to G proteins, GABA_B_R), expressed in oligodendroglial cell membranes participate in phenomena that promote myelination^1,2,5,6^. However, the development of specific drugs that act on oligodendrocyte receptors is still far from complete, this lack of knowledge affects not only the study of GABAergic signaling’s physiological role during oligodendroglial differentiation but also the development of drugs with clinical potential. The stimulation of GABAergic signaling during myelination should be done with specific drugs that act on the GABARs expressed in the oligodendroglial lineage. Current evidence indicates that GABA_A_R is involved in promoting or regulating myelination in different models^6,7^. The main GABA_A_R expressed in oligodendrocytes (OLs) has characteristics that distinguish it from receptors that are mostly expressed in neurons and astrocytes^3,8^. Thus, the GABA_A_R expressed in the membrane of oligodendrocyte precursor cells (OPCs) and mature OLs presents a combination that includes the α3 and γ1 subunits. The most likely composition corresponds to a α3β2γ1 receptor^8^. This receptor is potentiated by several β-carbolines, including n-butyl-β-carboline-3-carboxylate (β-CCB), which has a potent positive effect^3^. The binding site of various β-carbolines (including β-CCB) was originally described as that for benzodiazepines. However, other reports have proposed different binding sites such as that for loreclezole^9^. Our recent studies have shown that, in the case of oligodendroglial cells, β-CCB acts mainly as a positive modulator through a different binding site than that for benzodiazepines, either the classical binding site or the so-called low-affinity binding site^8^. Moreover, we have also shown that the action of β-CCB (and other β-carbolines) on the receptor expressed in cortical neurons in vitro is differential concerning the oligodendroglial receptor^5,8^. Thus, we hypothesized that β-CCB enhances oligodendroglial GABAergic communication in in vivo models^5^ and promotes the myelination process. β-CCB, was originally described as an inverse agonist of neuronal GABA_A_R, is a probable endogenous modulator of GABA_A_R expressed in the central NS (CNS)^10^. Systemically, β-CCB does not produce behavioral alterations, unlike other β-carbolines (e.g., DMCM, β-CCM) which act as convulsant agents and anxiogenics^10,11^. Indeed, β-CCB has no convulsant effect per se*,* even when it is administered to mice at doses of up to 100 mg/Kg^11^. In vitro experiments showed that β-CCB did not exhibit either potentiation or a robust effect as an inverse agonist on the GABA_A_ response of cortical neurons kept in culture^8^, supporting that observed in the whole animal.

We have previously shown that β-CCB treatment promotes remyelination in a focal demyelination model^5,12^. Here, we investigated whether the effect of β-CCB extends to other regions in the CNS. For this, we conducted a longitudinal study with MRI and a cross-sectional analysis with BGII specific histology and IF using the NG2^+^ and CC1^+^ oligodendroglial markers. Thus, we administered β-CCB to animals that were previously subjected to 0.3% CPZ in the food to induce myelin loss in the CNS, a well-known model for multiple sclerosis (MS)^13–15^, and tested whether β-CCB administration affected the rate and degree of remyelination. The results suggested that the myelin increase induced by β-CCB during the recovery period was not homogeneous across the different areas studied. Overall, β-CCB had a robust promyelinating effect in brain white matter (*corpus callosum*, *fimbria*, *internal capsule*, and *cerebellar peduncles*). This remyelination can be well monitored in some regions using MRI. In the cerebral cortex (*mctx*), β-CCB did not have a clear effect, whereas in some areas of the *hippocampus* it exhibited a mild effect. Thus, the promyelinating character of β-CCB was confirmed in a model of generalized demyelination, supporting the idea that β-carbolines have the potential to be used as therapeutic drugs against demyelinating diseases, as it has been shown elsewhere^12^, this promyelinating action could be explained by its potentiating effect on the oligodendroglial GABA_A_R.

## Results

### Cuprizone intake promotes alterations in white matter assessed by BGII staining and T2-weighted images

**Figure 1:**
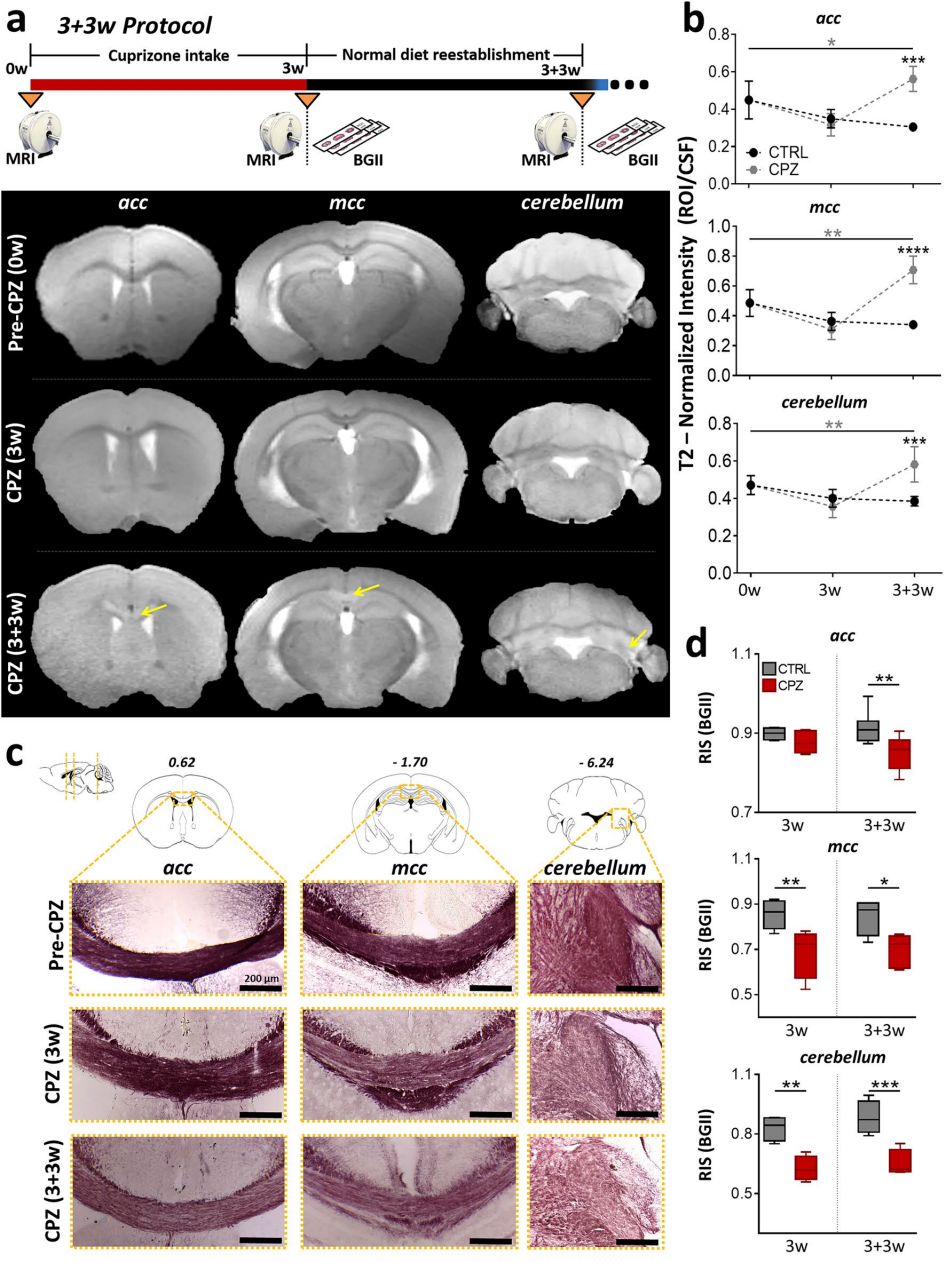
CPZ intake (0.3%) induces hyperintensity of the T2-wi signal in white matter that correlated with a decrease in BGII staining. (**a**) The top diagram illustrates the 3 + 3w protocol course, below, are shown tree representative T2-wi coronal images acquired before CPZ intake (0w in the diagram), after 3w of CPZ intake (CPZ (3w)), and after 3 subsequent weeks with normal diet reestablished (CPZ (3 + 3w)). Images illustrate the following areas of interest (yellow arrows): anterior *corpus callosum* (*acc*), medial *corpus callosum* (*mcc*), and cerebellar white matter (*cerebellum*). (**b**) Quantitative representation of longitudinal changes in normalized T2-wi signal intensity (ROI/CFS) estimated for each area analyzed. Gray asterisks correspond to longitudinal comparison within the CPZ group. ANOVA, n = 4 for control (CTRL) and n = 4–7 CPZ groups, respectively. (**c**) Brain coronal sections stained with BGII under the conditions shown in (**a**). Like in (**a**), the cerebral anterior (*acc*, + 0.62 mm from bregma) and medial (*mcc*, − 1.70 mm from bregma) sections are illustrated. The third column depict a cerebellar section (*cerebellum*, − 6.24 mm from bregma). (**d**) Relative intensity of staining (RIS) by BGII of *acc*, *mcc*, and *cerebellum* for control (3w; n = 4–5) and CPZ (3w; n = 4–7), and for control (6w; n = 4–7) and CPZ (6w; n = 4–7) samples. ANOVA, *P < 0.05, **P < 0.01, ***P < 0.001, ****P < 0.0001.

Several studies have shown that microstructural alterations in CPZ-demyelinated mice can be evaluated through T2-weighted images (T2-wi) in vivo, as the images show hyperintense areas that correlate with less myelin content^16,17^. Thus, this is a way to confirm the effect of CPZ intake and an obvious advantage when performing longitudinal studies^17,18^. Here, we first used T2-wi to detect hyperintensity in different white matter areas. We found that 0.3% CPZ intake for three weeks caused significant changes in the T2-wi signal intensity in all animals that ingested CPZ (n = 38), an effect that lasted for several weeks after mice were switched back to a normal diet. Microstructural changes were evaluated in CPZ-demyelinated animals subjected to CPZ for 3 weeks plus 3 subsequent weeks fed with normal diet (3 + 3w protocol) and comparing them versus the control (CTRL) group. T2-wi signal intensity was evaluated and compared in the anterior *corpus callosum* (*acc*, P = 0.0001), medial *corpus callosum* (*mcc*, P < 0.0001), and cerebellar white matter (*cerebellum*, P = 0.0007), which are commonly used to evaluate myelin content in several protocols^14,19–21^. As opposed to CTRL group, CPZ-demyelinated animals exhibited T2-wi signal hyperintensity in the three areas (Fig. 1a,b). This hyperintensity remained up to six weeks after initiating the intake. During this time frame, we also observed a ≈2% mortality rate in the CPZ intake group.

To verify if changes in T2-wi correlated with demyelinating damage, tissues from both the CPZ group and the CTRL group were divided and processed for BGII staining at two time points, first at the end of CPZ intake (3 weeks) and second at the end of the 3 + 3w protocol (Fig. 1c). Then, we evaluated the relative intensity of staining (RIS)^5,22^ and found that the 3w CPZ group presented a clear decrease in RIS for the *mcc* (P = 0.0057) and *cerebellum* (P = 0.0014), compared to the respective CTRL group (Fig. 1d). Also, at the end of the 3 + 3w protocol all three areas presented a significant decrease in RIS in the CPZ group, *acc* (P = 0.0047), *mcc* (P = 0.0195), and *cerebellum* (P = 0.0005) (Fig. 1d). Taken together, evidence suggested that in several regions CPZ intake caused T2-wi signal hyperintensity and a decrease in RIS observed through BGII staining, and that this consistently occurred as soon as 3 weeks after CPZ intake. Both effects were consistent with demyelination. All the animals that were included in subsequent experiments were tested during CPZ administration protocol to confirm the T2-wi hyperintensity changes in the described areas.

### CPZ intake induced alterations in diffusion tensor imaging metrics in cerebral and cerebellar white matter

To complement the longitudinal protocol, we conducted an MRI analysis using diffusion tensor imaging (DTI). DTI analysis has been widely used to describe white matter alterations. Here we used DTI to further detail the changes caused by CPZ intake. Fractional anisotropy (FA) index and radial diffusivity (λ_⊥_) ratio are two metrics calculated from this DTI analysis. Both metrics have been successfully used in experimental models to detect myelin abnormalities^5,23^. The apparent diffusion coefficient (ADC) was also calculated for each region. ADC is a parameter with an inverse pattern to that of the FA index. This provides robustness to the significance of the observed change^24^. Finally, the axial diffusivity (λ_||_) ratio was evaluated to obtain information about axonal integrity^25^.

**Figure 2:**
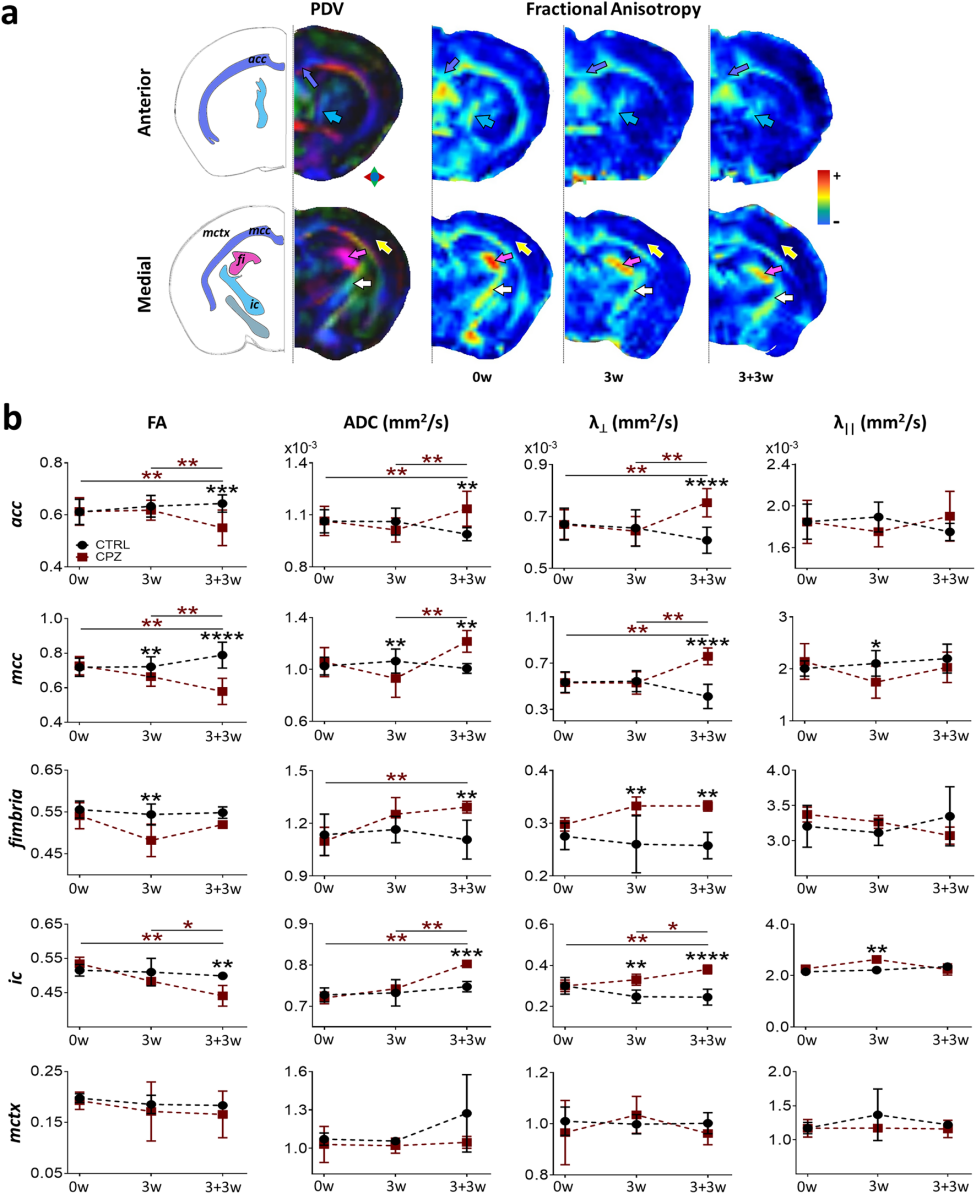
Diffusion tensor imaging (DTI) metrics for different brain structures of CPZ-demyelinated animals. (**a**) Principal diffusion vector (PDV) and fractional anisotropy (FA) maps during CPZ intake in anterior and medial brain coronal sections. FA maps were made before CPZ intake (Pre-CPZ; 0w), after 3 weeks of CPZ intake [CPZ (3w)], and after the subsequent three weeks with normal diet reestablished [CPZ (3 + 3w)]. Five areas of interest are indicated: *corpus callosum* (both *acc* and *mcc*), *fimbria*, *internal capsule* (*ic*), and *medial cortex* (*mctx*). (**b**) DTI metrics: FA, apparent diffusion coefficient (ADC), radial diffusivity (λ_⊥_), and axial diffusivity (λ_||_) ratio for the five areas depicted in (**a**) and were plotted Pre-CPZ (0w), CPZ (3w), and CPZ (3 + 3w) stages. Data represent the mean ± S.D. n = 4–16 for control group and n = 4–29 for CPZ-demyelinated group. Dark red asterisks correspond to a longitudinal comparison within the CPZ group, while black asterisks signal comparisons between groups. ANOVA. *P < 0.05, **P < 0.01, ***P < 0.001, ****P < 0.0001.

**Figure 3:**
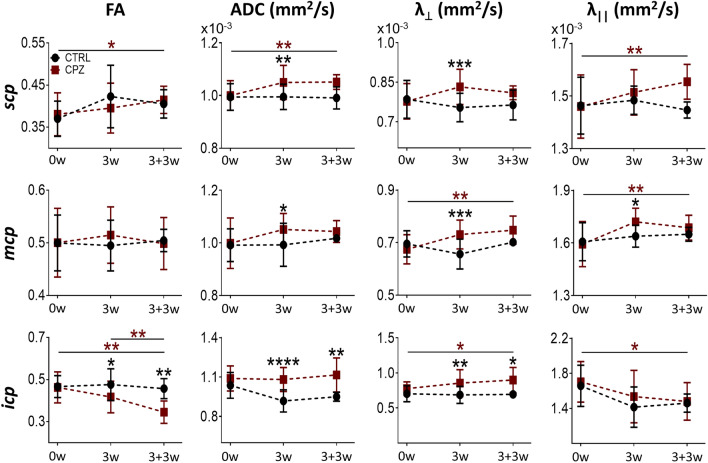
DTI metrics of *cerebellar peduncles* in CPZ-demyelinated animals. Graphics show the FA, ADC, λ_⊥_, and λ_||_ metrics for the superior (*scp*), medial (*mcp*), and inferior (*icp*) *cerebellar peduncle* of control animals (n = 4–16) and the CPZ-demyelinated group (n = 14–29) for the different CPZ stages: Pre-CPZ (0w), CPZ (3w), and CPZ (3 + 3w) as indicated. Data represent the mean ± S.D. Dark red asterisks correspond to a longitudinal comparison within the CPZ group, while black asterisks signal comparisons between groups (CPZ vs. control) using ANOVA. *P < 0.05, **P < 0.01, ***P < 0.001, ****P < 0.0001.

In this study, DTI metrics were evaluated for the *corpus callosum* (*acc* and *mcc*), *fimbria*, and the *internal capsule* (*ic*) in the cerebral white matter (Fig. 2). The superior (*scp*), middle (*mcp*), and inferior (*icp*) *cerebellar peduncles* were also evaluated (Fig. 3). Significant changes in DTI metrics were observed as early as 3 weeks after the start of CPZ ingestion, compared with the CTRL group. The FA index decreased in the *mcc* (P = 0.0028), *fimbria* (P = 0.0025), and *icp* (P = 0.0148) and these changes remained for up to three weeks after mice returned to a normal diet (3 + 3w protocol; *mcc*, P < 0.0001; *icp*, P = 0.0088). At 3 + 3w, we also detected significant differences in the *acc* (P = 0.0009) and *ic* (P = 0.0017) compared against the CTRL group. Regarding the ADC, after 3 weeks of CPZ intake, we observed a significant increase in the 3 *cerebellar peduncles* versus the CTRL group (*scp*, P = 0.0034; *mcp*, P = 0.0111; *icp*, P < 0.0001). These increases had a partial recovery at 3 + 3w for *scp* and *mcp*. Moreover, there was a significant increase in the ADC for the *acc* (P = 0.0012), *mcc* (P = 0.0017), *fimbria* (P = 0.0093) and *ic* (P = 0.0003) structures at 3 + 3w, compared to control animals, or even when values from the same CPZ group were compared versus the parameter before starting CPZ intake. Finally, λ_⊥_ ratio analysis showed a significant increase in the *fimbria* (P = 0.0018), *ic* (P = 0.0021), and the three *cerebellar peduncles* (*scp*, P = 0.0008; *mcp*, P = 0.0003; *icp*, P = 0.0015) after three weeks of CPZ intake, compared to the control group. At 3 + 3w, a significant increase was maintained in the *acc* (P < 0.0001), *mcc* (P < 0.0001)*, fimbria* (P = 0.0014)*, ic* (P < 0.0001)*,* and *icp* (P = 0.0182). Although there is a mild recovery for *mcp* and *scp* at 3 + 3w.

Taken together, the results indicated that CPZ intake induced alterations in MRI metrics, suggesting demyelination. These alterations appeared after the third week of continuous CPZ protocol. The data also support that demyelination continued even after drug administration was withdrawn (i.e., at 3 + 3w), a result that was consistent with previous reports^26–29^. A different result was obtained from the *mctx*, as DTI metrics did not present variations during CPZ intake (Fig. 2, lower panel). All analyses indicated, as expected, that both cerebral and cerebellar white matter structures were affected by CPZ intake (Figs. 2 and 3). Changes observed by DTI analysis were consistent with demyelination.

### β-CCB administration ameliorates the demyelination effect induced by CPZ intake

**Figure 4:**
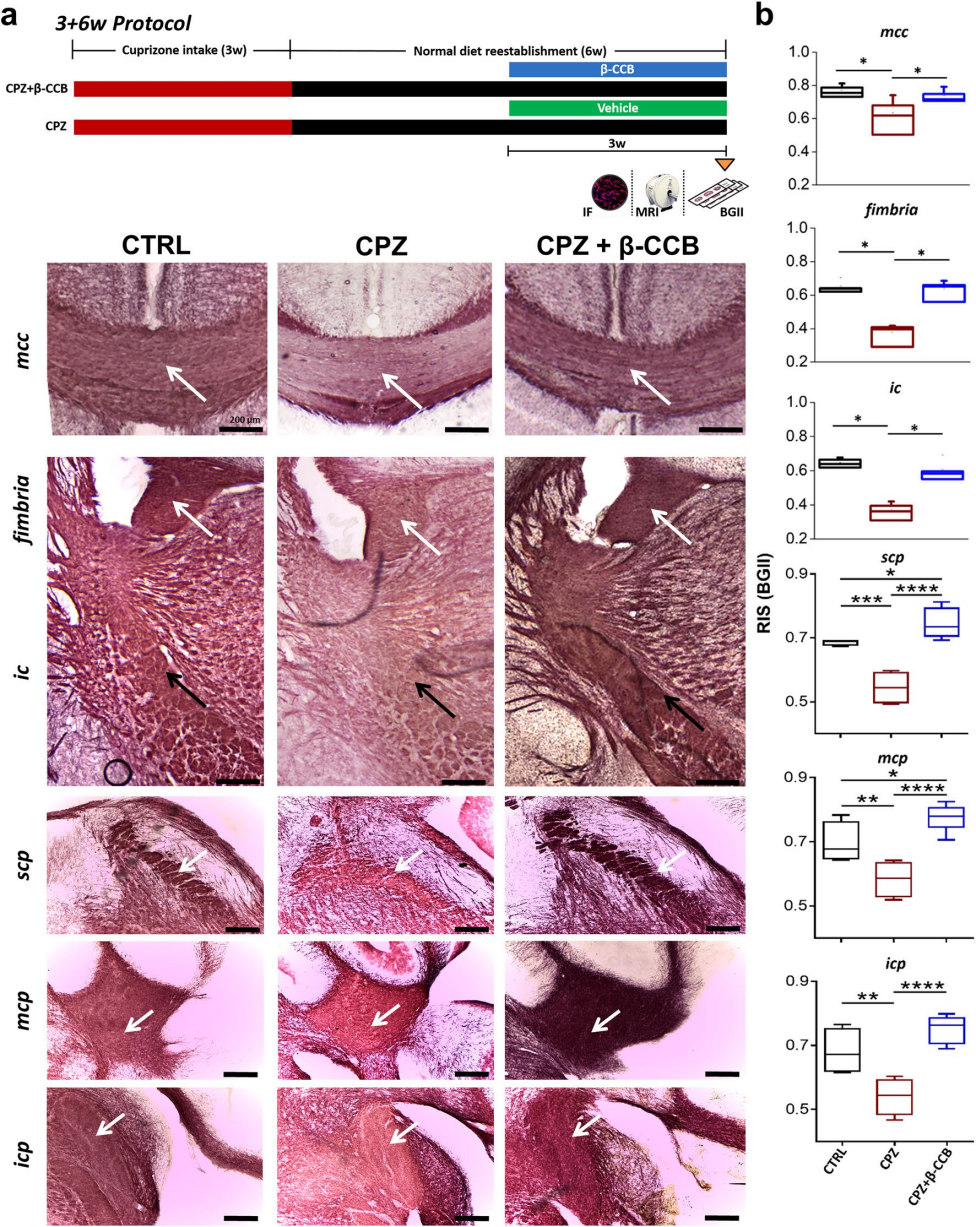
Histological BGII analysis of the β-CCB effect on brain and cerebellar white matter structures during CPZ intake. (**a**) The top diagram illustrates the 3 + 6w protocol course, below, images illustrate coronal slices in *corpus callosum* (*mcc*, white arrows), as well as in *fimbria* (white arrows), *internal capsule* (*ic*, black arrows), the superior (*scp*, white arrows), middle (*mcp*, white arrows), and inferior (*icp*, white arrows) *cerebellar peduncle* regions stained with BGII at the end of the 3 + 6w protocol course for a CTRL group that was fed with normal diet for 9 weeks (n = 4), CPZ group (injected with vehicle from week 6 to 9; n = 4), and the CPZ + β-CCB group that received β-CCB from week 6 to 9 (n = 4–7). (**b**) Box graphs represent the RIS evaluated for each condition as described in (a). Note that β-CCB in the CPZ + β-CCB group promoted an increase in RIS in all structures analyzed. ANOVA, *P < 0.05, **P < 0.01, ***P < 0.001. Each bar represents mean ± S.D.

**Figure 5:**
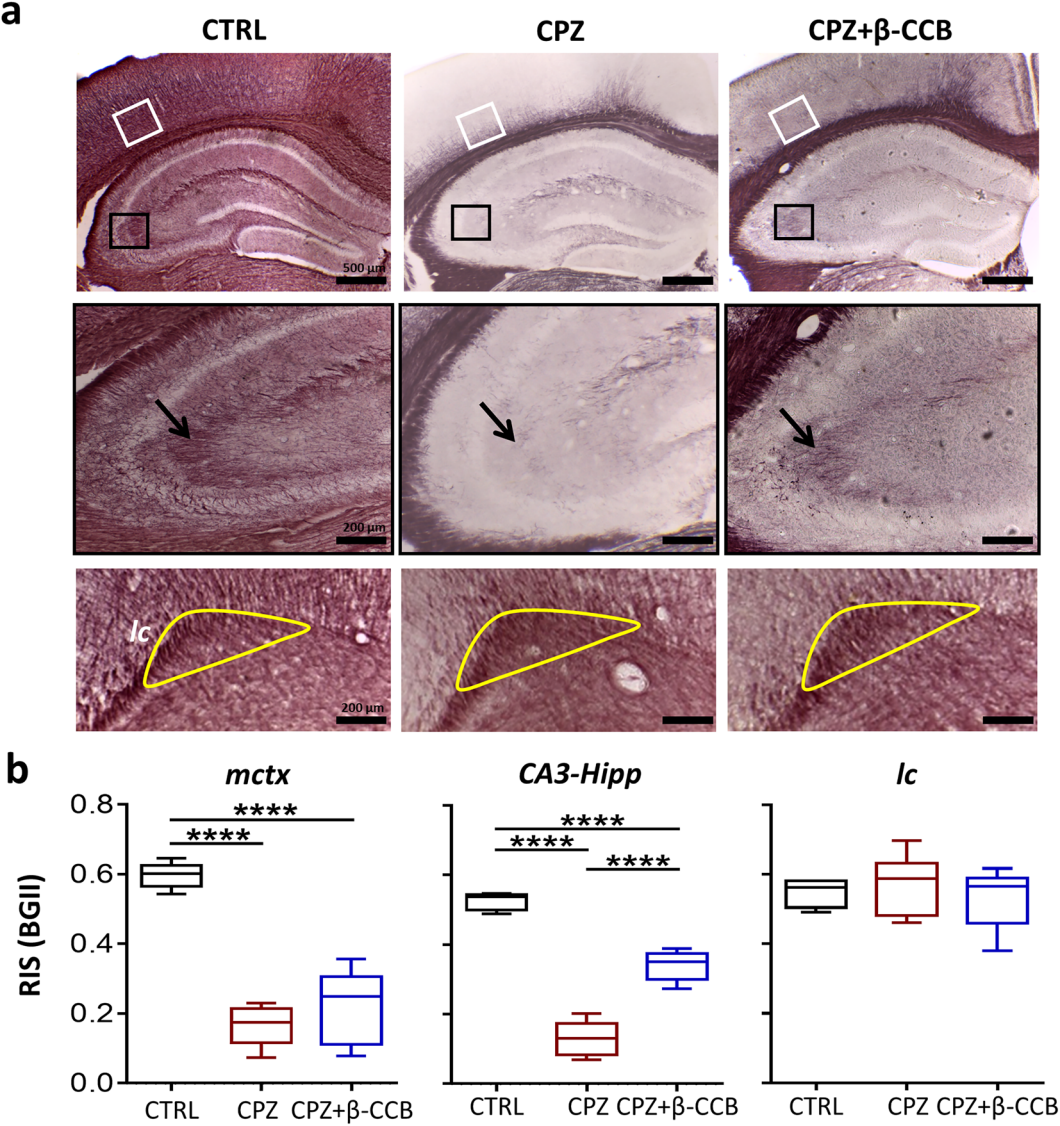
Histological BGII analysis of the β-CCB effect on brain gray matter and lateral cingulum in CPZ-demyelinated animals. (**a**) Images illustrate areas of the medial cerebral cortex (*mctx*, white box in top row), hippocampal *CA3* (*CA3*, black box in top row, amplified and signaled by black arrows in the central row), and lateral cingulum (*lc*, yellow area in bottom row); all images are in a coronal view. Samples were stained with BGII at the end of the 3 + 6w protocol following the diagram in Fig. 4a. Thus, CTRL group (n = 4–5) was fed with normal diet for 9 weeks, CPZ group (n = 5–6) was injected with vehicle from week 6 to 9, and CPZ + β-CCB group received 1 mg/Kg β-CCB (n = 5–6). (**b**) Box graphs represent the RIS evaluated for each condition. Note that β-CCB in the CPZ + β-CCB group promoted an increase in RIS only in the *CA3* area. ANOVA, ****P < 0.0001. Each bar represents mean ± S.D.

Previous studies have shown that β-CCB potentiates the response of GABA_A_Rs expressed in OPCs and OLs in culture, and that it has a promyelinating effect in a model of focal demyelination of the rat *icp*^3,5,8,12^. Identification of white matter structures that seem to undergo CPZ-induced demyelination, as assessed by MRI metrics and histology, allowed us to evaluate a generalized promyelinating effect of β-CCB. First, a histological analysis was realized for the areas illustrated in Fig. 4: *mcc*, *fimbria*, *ic*, *scp*, *mcp*, and *icp*. These areas were analyzed for the CTRL group, the CPZ group, and the CPZ group that was treated with β-CCB (CPZ + β-CCB). For the latter, β-CCB (1 mg/Kg) was administered by intraperitoneal injection from week 6 to week 9 in the 3 + 6w protocol, as indicated in the methods section, while the CPZ group that was not treated with β-CCB was injected with vehicle alone under the same administration protocol (see diagram in Fig. 4a). At the end of β-CCB treatment, samples from all three experimental groups were processed for BGII, and RIS was evaluated (Fig. 4a,b). Quantification showed that, for the CPZ group, RIS was significantly low compared to the CTRL group in all structures analyzed (*mcc*, P = 0.0466; *fimbria*, P = 0.0499; *ic*, P = 0.0487; *scp*, P = 0.0003; *mcp*, P = 0.0083; *icp*, P = 0.0028). However, we found that in the CPZ + β-CCB treated group, the RIS values were significantly higher than those estimated in the CPZ group (*mcc*, P = 0.0461; *fimbria*, P = 0.0410; *ic*, P = 0.04871; *scp*, P < 0.0001; *mcp*, P < 0.0001; *icp*, P < 0.0001). Moreover, RIS values of the CPZ + β-CCB treated group did not show statistical differences compared with those of the CTRL group, except for *scp* (P = 0.0386) and *mcp* (P = 0.0272). Thus, the histological evidence suggested that systemic administration of β-CCB had a promyelinating effect on the white matter that had been previously demyelinated by CPZ intake. Interestingly, no differences were observed in another white matter structure analyzed, the lateral cingulum (*lc*), that did not show differences when comparing the CTRL group, CPZ group, and the CPZ + β-CCB group (see Fig. 5b).

CPZ intake also generated significant demyelination in gray matter structures such as the *neocortex* and *hippocampus*^30^. Thus, we were interested in exploring these gray matter structures of significance for cognitive functions (Fig. 5) and relevant for pathologies such as MS. For this, experimental groups were treated as described above and then histologically analyzed by evaluating the RIS. The quantitative analysis (Fig. 5b) revealed that at the end of CPZ intake there was a profound reduction of RIS, suggesting that myelin content decreased in both *mctx* (P < 0.0001) and *hippocampus* (P < 0.0001). β-CCB administration did not appear to have a promyelinating effect on the *mctx*, as the RIS values did not improve in the CPZ + β-CCB group (P < 0.0001). Also, the effect of β-CCB on the *hippocampus* in general did not appear to be significant. However, by performing an analysis of distinct hippocampal substructures, we noted that β-CCB lead to an increase in RIS in the *CA3* area (*CA3*, P < 0.0001), however, RIS did not recover control levels, suggesting a limited promyelinating effect (Fig. 5b).

### DTI analysis of CPZ-demyelinated animals treated with β-CCB

The histological results suggested that β-CCB treatment promoted the recovery of CPZ-demyelinated animals, particularly in white matter regions. Thus, finding highly reliable areas of the nervous system concerning myelin content changes, reported by MRI, will be useful for developing a method to monitor these changes longitudinally. This would facilitate pharmacological testing with potential substances that enhance remyelination. It is well known that interpreting microstructural changes is limited under current technical imaging conditions. However, longitudinally tracking the process will stimulate the search for reliable protocols. The histological data confirm the promyelinating effect of β-CCB administration; therefore, the following MRI analysis was performed to determine the areas where DTI metrics can confirm this effect.

**Figure 6:**
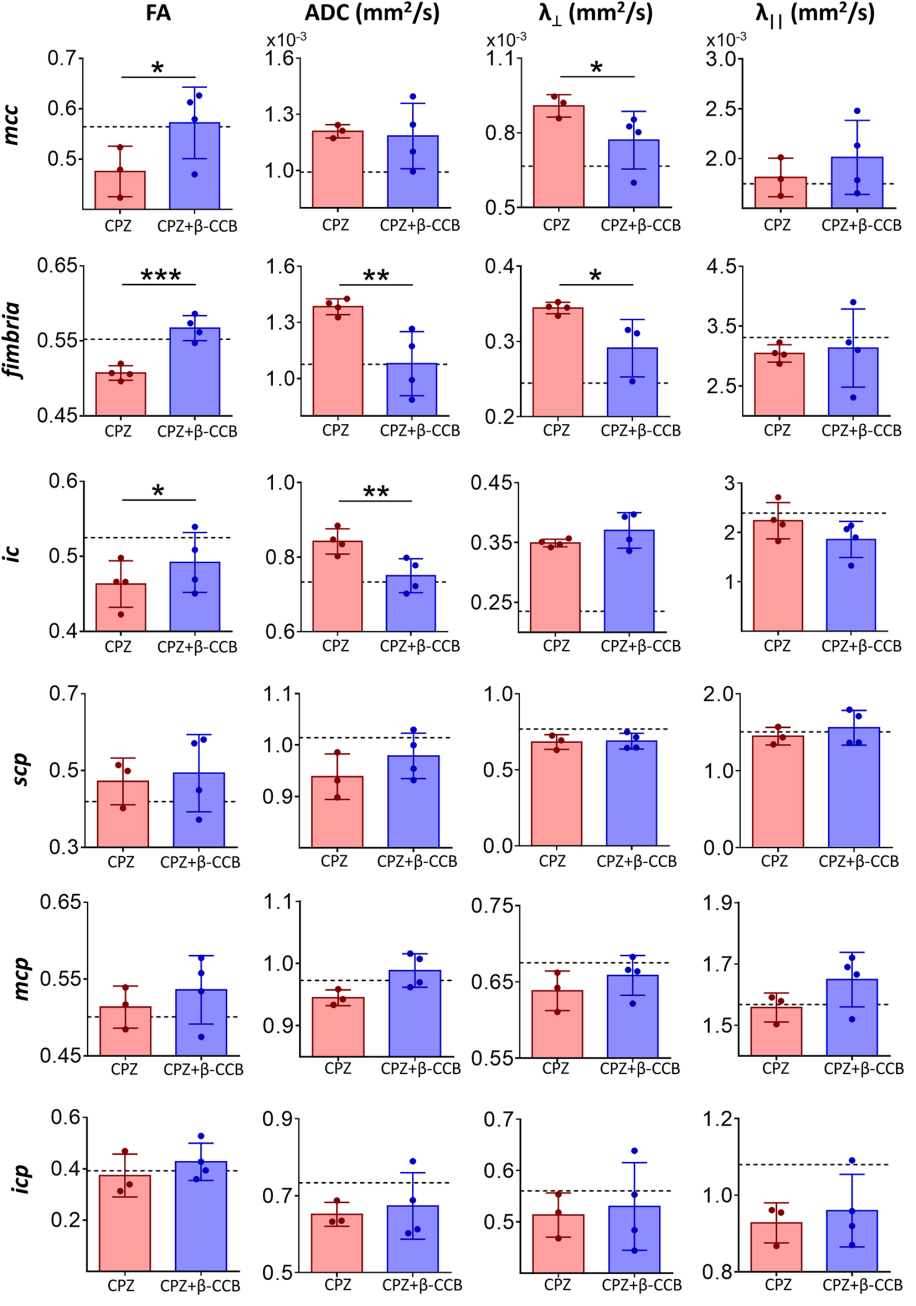
DTI metric analysis of the β-CCB effect on white matter structures in CPZ-demyelinated animals. Effect of β-CCB treatment on white matter metrics evaluated by MRI; bar graphs indicate mean ± S.D. (dotted lines represent the mean for the control group) for FA, ADC, λ_⊥_, and λ_||_ metrics obtained at the final stage of β-CCB administration for the *mcc*, *fimbria*, and *ic* regions and the three *cerebellar peduncles* (*scp*, *mcp*, and *icp*), and comparing the metrics computed for the CPZ group vs. the CPZ + β-CCB group. Every point in graphics is the value for a given brain analyzed (n = 3–4 for each group). Student's t-test. *P < 0.05, **P < 0.01, ***P < 0.001.

The bar graphs in Fig. 6 show the parameter values obtained by DTI analysis for all areas analyzed: *mcc*, *fimbria*, *ic*, and the three *cerebellar peduncles* (*scp*, *mcp*, and *icp*). DTI metrics were evaluated at the end of treatment with β-CCB and compared versus the CPZ group injected with vehicle. It was found that all *fimbria* values (FA, P = 0.0009; ADC, P = 0.0068; λ_⊥_, P = 0.0186) were consistent with the histological findings, while those for *mcc* (FA, P = 0.0442; λ_⊥_, P = 0.0471) and *ic* (FA, P = 0.0477; ADC, P = 0.0086) correlated partially (Fig. 6). Specifically, it was identified a significant increase in the FA index in animals treated with β-CCB or decreases in ADC and λ_⊥_ ratio values, all of which indicate a possible increase in myelin content. Finally, the λ_||_ ratio did not exhibit significant changes. Metrics analyzed for the *cerebellar peduncles* did not correlate with the histological findings. Thus, β-CCB showed a potential generalized promyelinating effect in the brain of CPZ-demyelinated adult animals as analyzed by BGII histology, however, these findings are only partially correlated with DTI metrics.

**Figure 7:**
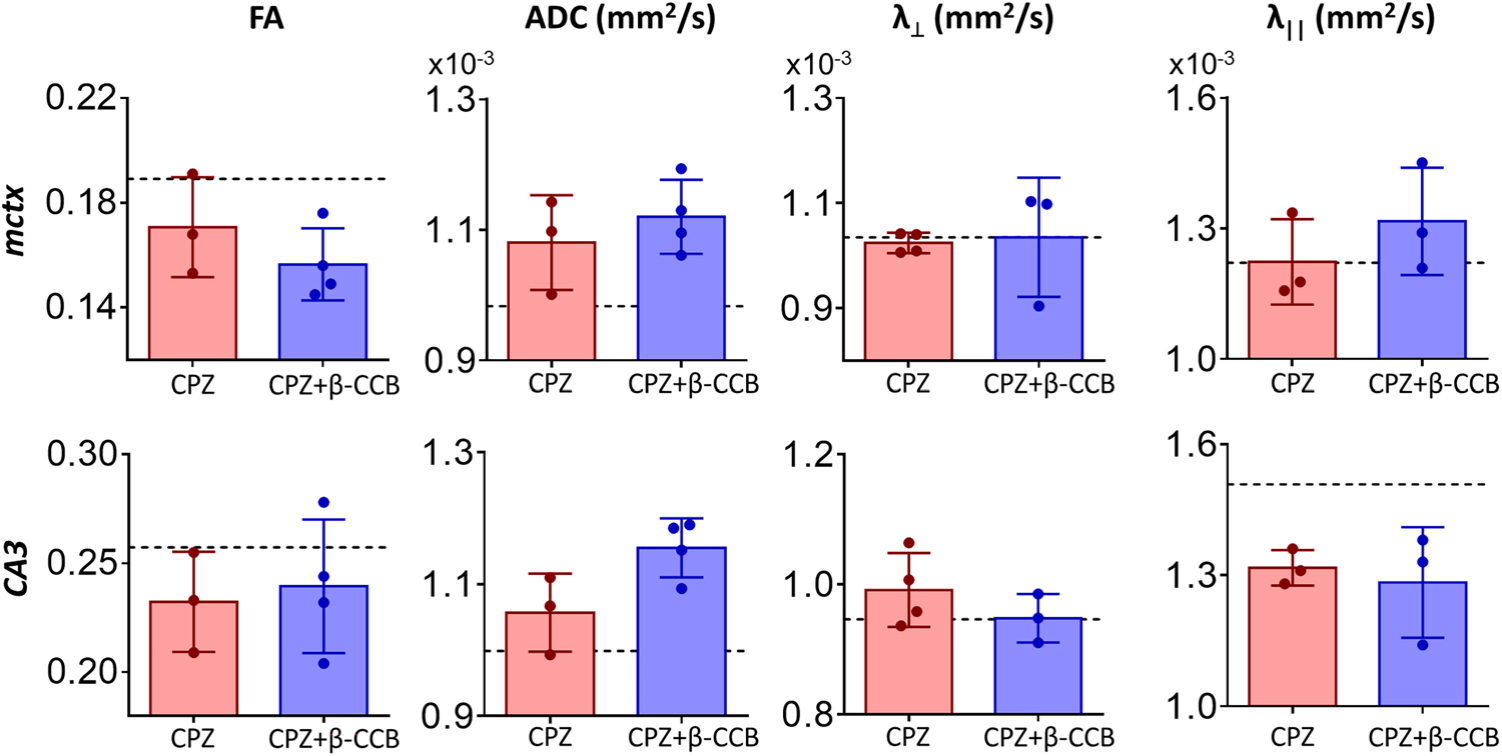
DTI metric analysis of the β-CCB effect on cerebral gray matter structures in CPZ-demyelinated animals. Effect of β-CCB treatment on cerebral gray matter metrics evaluated by MRI; bar graphs indicate mean ± S.D. (dotted lines represent the mean for the control group) for FA, ADC, λ_⊥_, and λ_||_ metrics obtained at the final stage of β-CCB treatment for the *mctx* and *CA3* regions, comparing the metrics computed for the CPZ group vs. the CPZ + β-CCB group. Every point in graphics is the value for a given brain analyzed (n = 3–4 for each group). Student's t-test.

On the other hand, DTI metric analysis indicated that myelin did not increase in the *mctx* (Fig. 7) in animals treated with β-CCB. This result supports the histological findings. It is important to mention, however, that DTI analysis for *mctx* did not show changes elicited by CPZ intake at 3 + 3w (see Fig. 2a). This result differs from that of the histological analysis (Fig. 5). In the case of *CA3* in the *hippocampus*, DTI metrics for the group treated with β-CCB did not show changes compared to the respective control. Thus, DTI parameters were inconsistent with the histological observations, particularly for regions of gray matter analyzed here.

### Analysis of oligodendroglial lineage cells after β-CCB administration in the CPZ model

**Figure 8:**
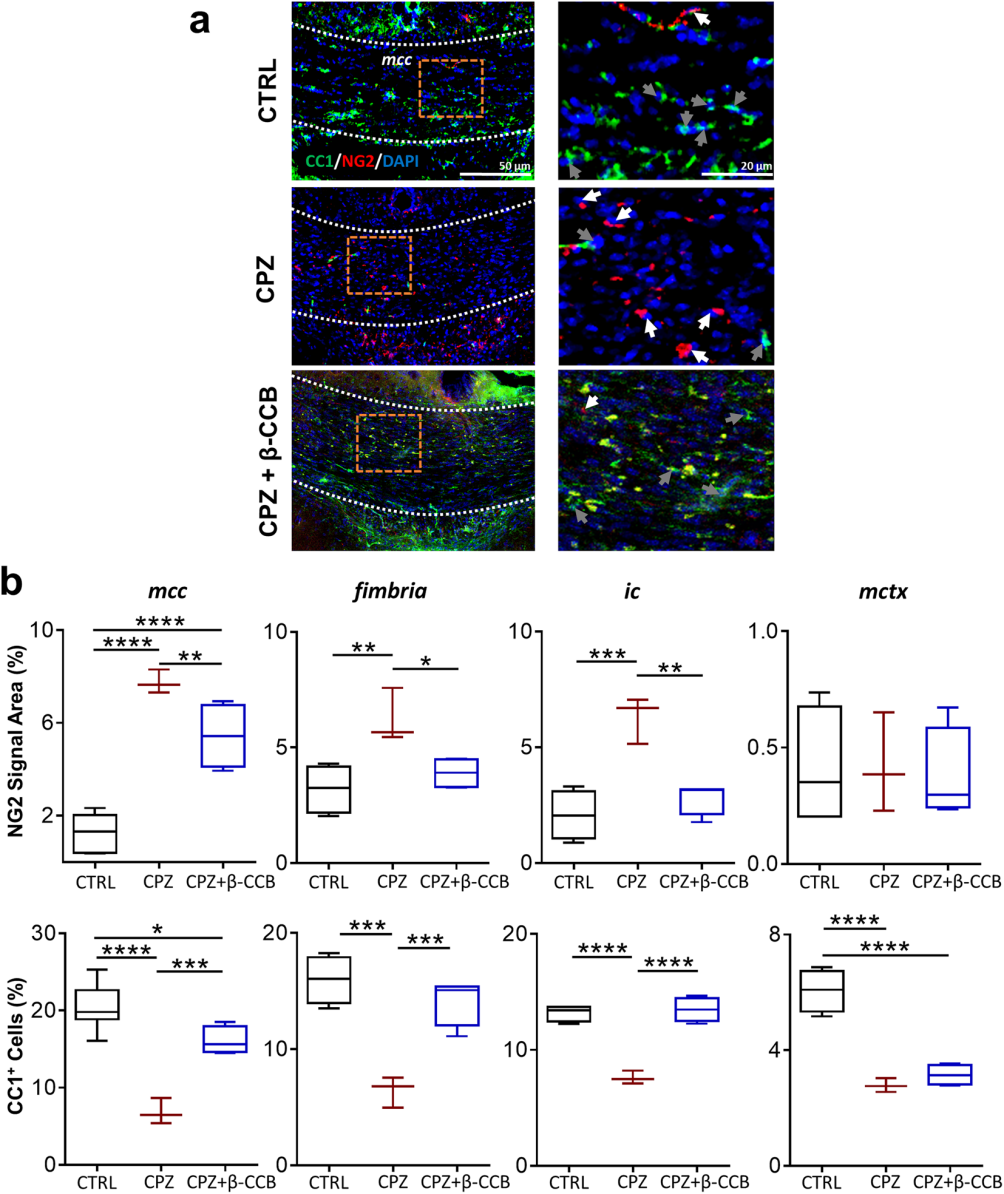
Effect of β-CCB on the oligodendroglial lineage population after CPZ ingestion. (**a**) Illustrative images of coronal sections of the *corpus callosum* (*mcc*) with CC1/NG2/DAPI labeling, showing panoramic micrographs (left) as magnifications (right) of the dotted boxes shown in the panoramic images. Samples were acquired for the CTRL group (n = 4–7), the CPZ group (n = 3), and the CPZ + β-CCB group (n = 4) as explained in Fig. 4. (**b**) Box graphs represent the area of NG2 (white arrows) signal and CC1^+^ (gray arrows) cells quantified for each condition as indicated and corresponding to the groups described in (**a**). ANOVA, *P < 0.05, **P < 0.01, ***P < 0.001, ****P < 0.0001. Each bar represents the mean ± S.D.

As mentioned above, β-CCB acts on GABA_A_R expressed in OLs and OPCs maintained in vitro, acting as a positive allosteric modulator^3^. It is also known that OPCs are the main source of remyelination, and GABAergic signaling could be involved in OPCs proliferation and maturation^6^. In this context, the promyelinating effect of β-CCB administrated in vivo may occur through GABA response potentiation in the oligodendroglial lineage (see also ref.^12^). Therefore, to identify a possible direct effect of β-CCB, we performed an immunolabeling analysis of OPC and mature OL populations using the NG2 and CC1 markers, respectively^1,31,32^ (Fig. 8a). For this, samples were processed at the end of β-CCB treatment to quantify either NG2^+^ signal and CC1^+^ cells for different cerebral white matter structures (*mcc*, *fimbria,* and *ic*) and one gray matter structure (*mctx*) in three experimental conditions: CTRL, CPZ group and CPZ + β-CCB (Fig. 8b). The results showed that the *fimbria*, *ic*, and *mcc* in the CPZ group presented a significant increase in the NG2^+^ signal (*fimbria*, P = 0.0033; *ic*, P = 0.0003; *mcc*, P < 0.0001) and a decrease in the number of CC1^+^ cells (*fimbria*, P = 0.0002; *ic*, P < 0.0001; *mcc*, P < 0.0001) compared to CTRL group. However, the CPZ + β-CCB group showed an important reversal of the latter pattern for both NG2^+^ signal area (*fimbria*, P = 0.0130; *ic*, P = 0.0011; *mcc*, P = 0.0093) and in the number of CC1^+^ cells (*fimbria*, P = 0.0007; *ic*, P < 0.0001; *mcc*, P = 0.0005), although the recovery observed did not reach control levels for the *mcc* (NG2^+^, P < 0.0001; CC1^+^, P = 0.0189). Finally, for *mctx*, CPZ intake did not produce significant changes in the NG2^+^ signal but induced a robust decrease in the number of CC1^+^ cells (P < 0.0001), a change that β-CCB was not able to revert (P < 0.0001).

Together, these results suggest that CPZ administration generated an increase in NG2^+^ cells in white matter structures but not in gray matter structures. It also caused a decrease in CC1^+^ cells in both white and gray matter structures. Finally, β-CCB treatment induced a reduction of the NG2^+^ cell population and a parallel increase in CC1^+^ cells in white matter. However, β-CCB did not show any significant effect on the oligodendroglial population analyzed in the *mctx*.

## Discussion

Here we showed that β-CCB acts as a promyelinating factor in different areas of the brain that were previously demyelinated by the CPZ intake protocol. It was shown that the promyelinating effect of β-CCB can be transversely tracked with specific histology by BGII or IF. Furthermore, changes in the demyelination-remyelination process can be followed through longitudinal analysis with MRI.

This information could help to develop experimental protocols for longitudinal monitoring of the myelinating process and to study the mechanisms activated by molecules that promote the process. Eventually, this will boost the development of novel therapeutic strategies for the treatment of demyelinating illnesses.

There are multiple demyelinating pathologies, each one with its unique characteristics and causal agents. An example of these pathologies is MS that is a demyelinating pathology of the CNS that affects both white matter and gray matter^33,34^. The CPZ intake model is a widely used experimental approach that produces characteristics resembling those of MS, and it allows researchers to evaluate the processes of demyelination and remyelination^13,14,35^. In this work, we used the CPZ model to induce changes in myelin content under a three-week administration protocol^26–30,36–43^.

We determined that 0.3% CPZ for three weeks caused substantial changes in myelin content indicators. To reach this conclusion, we assessed the myelin changes with different techniques, the first of which was T2-wi. This MRI technique has been used to study the evolution of MS through changes in lesion volumes^44^. Hyperintensity changes revealed by T2-wi provided a profile of possible areas suffering from demyelination. Thus, all animals included in the CPZ group were validated using T2-wi analysis. However, changes in hyperintensity can also be caused by inflammation and edema^45,46^, so a more detailed analysis is required to determine the possible causes of the observed changes (see^47^). Through our T2-wi analysis, we found that the CPZ protocol in mice, caused clear hyperintense areas in the *corpus callosum* and the white matter of the *cerebellum*. To confirm that these areas showed decreased myelin content, brain samples from these animals were processed with the BGII myelin-specific staining method^48^. We found that areas that showed hyperintensity in the T2-wi analysis coincided with brain regions that showed a significant decrease in BGII staining. This finding suggests that both methods highlighted demyelination as a probable cause of the observed changes. Thus, at least for these two brain areas, T2-wi can be useful to quickly evaluate the action of experimental agents that affect myelination such as short periods of CPZ intake.

In some structures, diffusion magnetic resonance imaging (dMRI) was necessary in addition to T2-wi in order to evaluate different parameters that together provided more specific information on the microstructure of myelin in the CNS^23,25,49,50^. Before proceeding with this analysis, we examined the areas that had been demyelinated by CPZ using BGII staining and, more importantly, we confirmed the promyelinating effect of β-CCB in several brain regions. It has been previously described that β-CCB has a promyelinating effect on focal demyelination in the rat *icp*^5,12^. Here, we show that the β-CCB treatment in mice demyelinated by CPZ intake promotes the recovery of myelin content in various structures. This finding clearly indicates that the possible promyelinating effect of β-CCB is more generalized in the NS.

The significant demyelination of gray matter structures in the CPZ model resembles that of MS patients^30,33^. Hence, we decided to analyze two gray matter structures: the *mctx* and the *hippocampus*. Histological findings showed that CPZ caused a clear decrease in BGII staining intensity in both structures but it was also observed that β-CCB treatment did not seem to cause myelin recovery. Nevertheless, when performing an analysis of hippocampal substructures, we found that β-CCB seemed to promote the partial recovery of BGII staining that was restricted to the *CA3* area, suggesting a focalized promyelinating effect and the involvement of context-dependent factors. It is important to point out that histological analysis by BGII showed that the lateral cingulum (*lc*) did not present a decrease in myelin induced by CPZ intake, this confirmed results previously reported in this regard^51^. Furthermore, we did not observe β-CCB-induced changes in the *lc*. This supports the idea that the changes caused by CPZ and the lack of effects on the gray matter of the CPZ + β-CCB group represented reliable results, as the *lc* area acted as a sort of staining control.

In general, histological studies confirmed that in various areas of cerebral and cerebellar white matter, treatment with β-CCB promoted a recovery of myelin content evaluated by the intensity of BGII staining, suggesting that β-CCB acts as a general promyelinating agent in various tracts of the CNS.

It was therefore important to use MRI to evaluate the changes resulting from β-CCB treatment (CPZ + β-CCB group). This was done at the end of the trial with β-CCB and was important to determine whether the metrics obtained would support the histological results when these were compared with the CPZ group. First, we analyzed the *cerebellar peduncles*. Previous research has shown that these tracts are also affected by CPZ as well as in MS patients^52,53^. The metrics obtained by DTI analysis did not show significant changes between both groups; thus, the calculated metrics were not indicative of an improvement in myelin content and did not support the results obtained with histology. In contrast, when the *mcc*, *fimbria*, and *ic* areas were evaluated by DTI, diverse changes in metrics supported significant differences between the CPZ + β-CCB group and the CPZ group. In particular, we observed that metrics differed in the *fimbria* of these two groups. These differences were consistent with a promyelinating effect in the CPZ + β-CCB group. For example, the increase in FA in parallel with the significant decrease in the ADC and λ_⊥_ ratio are metrics consistent with an increase in myelin content, clearly supporting the idea of a promyelinating effect produced by β-CCB. Moreover, the increase in FA in both the *mcc* and *ic* indicated a possible increase in myelin content, which was further supported by a decrease in the λ_⊥_ ratio and ADC in the *mcc* and *ic*, respectively. Finally, the metrics evaluated for *mctx* and the *CA3* area did not show significant changes. However, as mentioned, β-CCB treatment did not cause an increase in BGII staining in the *mctx*.

The contrast between DTI metrics and the histological analysis when comparing the CPZ and CPZ + β-CCB groups could have several explanations, and more experiments will be needed to determine the causes. However, this study indicates that white matter structures like the *corpus callosum*, *ic,* or *fimbria* were consistent in several DTI metrics and histological data. In the case of the *fimbria*, this is of interest because previous studies using different intoxication protocols showed that CPZ (0.2–0.25%) had a partial or no effect on this structure^30,54^. In our protocol, CPZ (0.3% for three weeks) produced significant demyelination, as suggested by DTI and histological examination, this difference is more probably due to the higher dose used here.

BGII staining showed a clear effect of β-CCB on myelin enhancement in several regions, although when analyzed with MRI there was correlation with some areas while not with others (e.g., *cerebellar peduncles*). This could be explained either due to a low technical resolution or due to specific concomitant changes (e.g., inflammation) resulting from the demyelination-remyelination process in different areas. Thus, areas that exhibit edema, inflammation, or other alterations could interfere with an adequate evaluation of DTI metrics, as has been discussed elsewhere^55,56^. Interestingly, in previous studies carried out in rats^5,12^, the demyelination-remyelination process was followed effectively in the *icp* region with both techniques. However, since the present study used mice, the technical characteristics of MRI protocols are substantially different. In addition, focal demyelination process and systemic demyelination caused by CPZ surely present significant differences in terms of the cellular responses provoked by either method.

In this study we showed that β-CCB had no effect on gray matter regions such as the *mctx*, as determined by both BGII and MRI. However, it is clear that CPZ had a significant effect on myelin content, as this has been documented before^30,57,58^. Similarly, no significant changes were observed in the *hippocampus* of the β-CCB-treated group, yet in the *CA3,* β-CCB-treatment seemed to partially reproduce the positive effect observed in other areas. One possible explanation for the lack of effect is a divergence in β-CCB action mechanisms at different areas of the CNS. Differences are expected due to ample evidence of genotypic and functional diversity of oligodendroglial cells^59–61^ and especially of the expression of GABA_A_R with different molecular compositions^62^, but also due to differences in the specific environmental contexts of each area^63^. These difference could determine the interaction between oligodendroglial cells and distinct cell types, mainly astrocytes and neurons^64–67^. In this sense, the divergence in regulatory remyelination mechanisms is dependent on intrinsic and extrinsic factors as well as epigenetic mechanisms^68–71^.

The promyelinating effect of β-CCB might be related to its potentiating effect on the response to GABA in cells of the oligodendroglial lineage^3,5,8,12^. It is known that OPCs are the main source of remyelination, where GABAergic signaling could be involved in OPC migration, proliferation, and differentiation processes^6^. In OPCs, GABA_A_R activation causes depolarization that activates voltage-gated channels with a consequent influx and increase in cytosolic Ca^2+^. It is also known that intracellular Ca^2+^ increase is involved in OPC proliferation, migration, and differentiation to myelinating cells^72,73^. Thus, potentiation of oligodendroglial GABAergic signaling induced by β-CCB might promote the activation of this cascade, resulting in OPC differentiation to mature OLs^12^. Quantitative immunolabeling analysis of OPCs and OLs using NG2 and CC1 markers indicated that CPZ intake caused a significant increase in the number of NG2^+^ cells and a decrease in CC1^+^ cells in white matter structures. These changes in the dynamics of oligodendroglial lineage populations were reversed by systemic treatment with β-CCB. In this way, the decrease in the number of NG2^+^ cells could be due to the fact that β-CCB enhanced the GABA response in precursor cells that eventually would promote their maturation to OLs, as suggested by the increase in CC1^+^ cells. The CPZ intake did not generate an increase in NG2^+^ cells in the *mctx*, but a clear decrease in CC1^+^ cells was found. This result was consistent with a demyelination process evidenced by BGII staining. Low OPC density, even after CPZ intake, might explain an inefficacy in generating new myelinating OLs in the *mctx*. However, more experiments are needed to clarify the process underlying this phenomenon.

Finally, it is well known that determining the content of myelin in the gray matter is complicated using DTI analysis^74^. This effect was reflected when comparing *mctx* metrics obtained from the CTRL group and the CPZ-demyelinated group, where the metrics did not suggest a decrease in myelin, even though histology showed clear images consistent with demyelination. In this case, more advanced methods for image acquisition and analysis are necessary to study gray matter areas. A final hypothesis to explain these discrepancies in gray matter structures is that CPZ intoxication is known to cause neuronal death, which would lead to axonal degeneration and an irreversible loss of myelin^75,76^. More studies are required to assess whether this deleterious effect could also explain the lack of β-CCB effects in the gray matter.

In summary, our results indicated that 0.3% CPZ administered for three weeks in the diet induced demyelination in mice. This brief protocol allowed for a better management of experimental groups with low animal mortality. It also allowed us to track the demyelinating and remyelinating stages of the model with the main purpose of evaluating the promyelinating effects of drugs with therapeutic potential. Here, we show that β-CCB, a positive allosteric modulator of the oligodendroglial GABA_A_R, has a robust promyelinating effect in different cerebral and cerebellar areas inducing an increase in the population of CC1^+^ mature oligodendrocytes.

## Materials and methods

### Study approval

All experiments were performed according to the procedures approved by the Ethics Committee of the Instituto de Neurobiología, Universidad Nacional Autónoma de México. Experiments were performed in C57BL/6J wild-type male mice (8 weeks old) following the Guide for the Care and Use of Laboratory Animals (National Institute of Health). Animals were kept under conventional housing conditions (22 ± 2 °C, 55 ± 10% humidity, 12 h day/night cycle and with ad libitum access to food and water) at the Instituto de Neurobiología, Universidad Nacional Autónoma de México. All possible efforts were made to minimize animal suffering and the number of animals used, and procedures complied with ARRIVE guidelines.

### Experimental design

Animals in the control (CTRL) group were fed with normal diet for either 3, 6, or 9 weeks. To induce demyelination, a group of animals were fed with food containing 0.3% CPZ for 3 weeks and subsequently switched back to normal diet for 3 (3 + 3w protocol; Fig. 1a) or 6 (3 + 6w protocol; Fig. 4a) more weeks. 3 + 6w groups were injected intraperitoneally the last 3 weeks of the protocol either with vehicle alone (CPZ group) or, as this is detailed below, were injected with β-CCB (CPZ + β-CCB). Myelin content was evaluated at 4 points: (1) Before CPZ ingestion (0w; using MRI), (2) After 3 weeks of CPZ intake (3w; using both MRI and BGII), (3) At the end of 3 more weeks under normal diet (3 + 3w protocol; MRI and BGII), and (4) After 3 subsequent weeks in which animals were either treated with β-CCB or injected with vehicle alone (3 + 6w protocol; MRI and BGII). Additionally, at point 4, cell populations of the oligodendroglial lineage were analyzed by IF.

### Cuprizone-induced demyelination

The animals had an acclimatization period to the experimental location for 1 week before beginning the study^38^. Experimental demyelination was induced by feeding 8-week-old male C57BL/6J mice with a diet containing 0.3% CPZ^39,42^ [bis(cyclohexanone)oxaldihydrazone] mixed with standard food (Specially Formulated Prolab, Laboratory Animal Diet. LabDiet) for three weeks^26–30,36–43^ and subsequently reestablishing the normal diet, for either three (3 + 3w protocol) or six weeks (3 + 6w protocol) depending on the experiment, a period in which the demyelination process continues^26–29^.

### β-CCB administration

For in vivo β-CCB injection, aliquots of β-CCB solution were prepared as treatment and vehicle solution as a control. Every day before injection, 1 mg of β-CCB was diluted in DMSO and saline (2%/98% v/v) mixture (saline was 0.9% NaCl), whose volume was 1 mL per aliquot. The mixture was sonicated for 60 min at 37 °C. Then, vehicle alone (injected to CPZ group) or 1 mg/Kg β-CCB^5,12^ (injected to CPZ + β-CCB group) solution was intraperitoneally (i.p.) administered. Either injection was given every 24 h for three more weeks after six weeks of beginning the experiment (i.e., 3w CPZ intake and then reestablishing normal diet at the end of the third week), to complete a 3 + 6w protocol (see diagram in Fig. 4a). Mice were randomly assigned to each treatment based on a blinded analysis. In all cases, β-CCB treatment did not cause obvious alterations in animal behavior (e.g., anxious-like behavior).

### Magnetic resonance imaging

All experimental groups were scanned at four times points: (1) Before CPZ ingestion, (2) After 3 weeks of CPZ intake, (3) At the end of 3 more weeks under normal diet (3 + 3w protocol), and (4) After 3 subsequent weeks in which animals were either treated with β-CCB or injected with vehicle alone (3 + 6w protocol). Anesthesia was induced with 2% isoflurane (in compressed air) using an anesthetic chamber and then maintained under 0.8–1% with a facemask during the procedure. T2-wi and diffusion magnetic resonance imaging (dMRI) were performed at the Laboratorio Nacional de Imagenología por Resonancia Magnética (LANIREM) using a 7 T magnet (Bruker Pharmascan 70/16US), interfaced to a Paravision 6.0.1 console (Bruker, Ettlingen, Germany), and with a Helium-cooled two-channel rat-head coil (Bruker Cryoprobe). DTI data sets were acquired using a spin-echo, single-shot echoplanar imaging sequence with the following parameters: slice thickness = 0.35 mm, slice gap = 0.1 mm, no physiological gating, RT = 2.23 s, ET = 23.4 ms, FOV = 28 × 28 mm^2^, image size = 150 × 150 mm, 40 diffusion directions, two different b values: 650 and 1250 s/mm^2^, 1 average, 46 slices, and with a total scanning time of 19 min, 19 s and 27 ms. T2-wi was acquired with the following parameters: slice thickness = 0.5 mm, inter-slice gap = 0.15 mm, RT = 4.2 s, ET = 33 ms, FOV = 30 × 15 mm^2^, image size = 256 × 256 mm^2^, rare factor = 8, 2 averages and 25 slices, and with a total scanning time of 4 min and 48 s. Pre-processing of dMRI data sets included both reduction of motion and eddy current-induced geometric distortions by linear transformation of each volume to the average non-diffusion weighted volume and denoising via random matrix theory^77^. The MRtrix software package (http://www.mrtrix.org) was used to estimate the tensor model, from which we derived fractional anisotropy (FA) maps, principal diffusion vector (PDV), radial and axial diffusivities (λ_⊥_ and λ_||_, respectively), and the apparent diffusion coefficient (ADC). Region of interest (ROI) of *acc*, *mcc*, *ic*, *fimbria*, *cerebellum*, *scp*, *mcp*, *icp*, *CA3* of the *hippocampus*, and *mctx* were manually delineated on PDV images. DTI parameters were calculated using ROI analysis.

### Black-Gold II staining

For histological examination, mice were transcardially perfused with 0.1 M phosphate-buffered saline (PBS) (pH 7.4) followed by 4% paraformaldehyde (PFA) in the same buffer; subsequently, the brains were dissected. Then, to cryopreserve them, they were placed in a PBS 30% sucrose solution (50 mL PBS/15 g sucrose) for 24 h. After cryopreservation, coronal sections with a thickness of 50 µm were obtained in a cryostat (Leica CM 1850) at a temperature between – 24 °C and – 26 °C and suspended in PBS. BGII staining, a myelin-specific aurohalophosphate compound^48^, was performed in suspension for the evaluation of myelin content^5,12^. For this, each 50 µm section was rinsed with ultrapure water for 2 min, then incubated in BGII solution (dissolved at 0.3% in 0.9% NaCl solution) for 12–20 min at 60–65 °C. Subsequently, the sections were rinsed with PBS for 2 min and then with 1% sodium thiosulfate (Na-Thio) for 3 min at 60–65 °C, and finally a series of two rinses with PBS for 2 min each. Then, sections were placed on slides and xylol was added for 1 min. Subsequently, DPX medium was added and a coverslip was placed. For myelinated zone assessment, tissue sections for BGII were visualized and analyzed under a microscope, and representative images were acquired with a Leica ICC50 HD camera (Leica Microsystems, Wetzlar, Germany). The relative intensity of myelin BGII staining (RIS) in selected brain areas was quantified using ImageJ software (version 1.52i). After converting the images to grayscale, the relative intensity was obtained from a given ROI, calculated by normalizing intensity values from each ROI against the background intensity value from each section^22^, applying the following relationship: RIS = (intensity of background − mean intensity of labeling in ROIs)/intensity of background.

### Immunofluorescence

To examine cell populations of the oligodendroglial lineage, specifically OPCs and OLs, 50 µm thick coronal sections were immunoassayed with antibodies against oligodendroglial cell-specific markers: mouse anti-APC/CC1 (1:250; Sigma-Aldrich, OP80) for mature OLs^1,31,32^ and rabbit anti-NG2 (1:250; Millipore, AB5320) for OPCs^1,32^. First, the fixed sections were washed and permeabilized in PBS with 0.1% Tween-20. Next, the sections were incubated in a blocking buffer solution (PBS, 5% BSA, and 0.3% Triton X-100) for 30 min at 4 °C. They were then incubated with the primary antibodies (diluted in PBS containing 5% goat serum and 0.1% Tween-20) overnight at 4 °C. Sections were then rinsed in PBS with 0.1% Tween-20 and incubated for 4 h at 4 °C with one of the following Alexa fluorophores-conjugated: 1:300 goat anti-mouse IgG (H + L) conjugated to Alexa Fluor 488 (Invitrogen, A-11001) or 1:300 goat anti-rabbit IgG (H + L) conjugated to Alexa Fluor 594 (Abcam, ab150080) depending on the host species of the primary antibodies. To identify the cell nuclei, five washes were performed in PBS with 0.1% Tween-20, and the sections were subsequently stained with 4′,6-diamidino-2-phenylindole dihydrochloride (4 μg/mL DAPI, Molecular Probes, D1306) and mounted on Mowiol (Sigma-Aldrich, 81381). In all cases, to corroborate the absence of nonspecific interactions of the secondary antibodies, the primary antibodies were omitted. Finally, the preparations were visualized under an LSM880 laser scanning confocal microscope (Zeiss, Oberkochen, DE). Each slice was then observed using a 20 × objective and images were acquired with a Z step size of 1 μm, forming a 3 × 1 tile-scan mosaic. Visualization and quantitative analysis of each Z-stack was performed using the NIH (USA) ImageJ software (https://imagej.net) and regions of interest (ROI) were outlined using the freehand selection tool. In the ROIs, the proportional area corresponding to the mark of NG2^+^ cells was estimated with respect to the total area of the ROI and in the case of the mature OLs, the proportion of CC1^+^ cells was estimated with respect to the total number of nuclei using the “Analyze Particles” plugin from ImageJ (https://imagej.net/imaging/particle-analysis). For each marker analyzed, it was considered as positive immunoreactive cell those that stand out from the background. The values were expressed as % of the total.

### Substances

β-CCB was obtained from Tocris Bioscience (Bristol, UK) and isoflurane from PiSa Lab (Guadalajara, JAL, México). All salts, PFA, CPZ and xylenes were acquired from Sigma-Aldrich (St. Louis, MO, USA). The BGII myelin staining kit was acquired from Millipore (EMD Millipore Corp., Billerica, MA, USA), and DPX mounting medium was from Fisher Scientific (FS, Fair Lawn, NJ, USA).

### Statistical analysis

All data are expressed as mean ± S.D. The means of two groups were compared using a Student's t-test or, when appropriate, an analysis of variance (ANOVA) followed by post-hoc comparisons of individual means using the Fisher correction. Animals were randomized to treatment according to local practice in participating centers. We assumed an effect size of around 1.4 (Cohen's d estimate calculation) and 80% power in detecting changes between group means in the in vivo experiments with a significance level of 0.05, which is consistent with previous studies using similar experimental models^5,16,58^. Homoscedasticity was verified using Levene's test. The Kolmogorov–Smirnov test was used to verify the normal distribution of the data. Statistical analysis was performed using GraphPad Prism software. Differences were significant at P < 0.05 (the exact values for P were indicated in the corresponding results section).

## Data Availability

The datasets used and/or analyzed during the current study are available from the corresponding author on reasonable request.
